# Gallbladder polyps – a follow-up study after 11 years

**DOI:** 10.1186/s12876-019-0959-3

**Published:** 2019-03-18

**Authors:** Linda Heitz, Wolfgang Kratzer, Tilmann Gräter, Julian Schmidberger, for the EMIL study group, G. Adler, A. Armsen, H-M Banzhaf, M. Bauerdick, P. Bernhardt, U. Bertling, B. O. Boehm, B. O. Brandner, S. O. Brockmann, M. Deckert, C. Dingler, S. Eggink, M. Fuchs, W. Gaus, H. Goussis, B. Gruener, A. Gruenert, M. M. Haenle, W. Hampl, C. Haug, B. Hay, L. Heitz, M-L Huetter, N. Iftikhar, A. Imhof, T. Kaltenbach, P. Kern, P. Kimmig, A. Kirch, D. Klass, W. Koenig, W. Kratzer, M. Kron, B. Manfras, K. Meitinger, T. Mertens, R. Oehme, G. Pfaff, I. Piechotowski, S. Reuter, T. Romig, A. F. A. von Schmiesing, S. Stanosek, G. Steinbach, M. Tourbier, A. Voegtle, T. Walcher, S. Wolff, P. Schlingeloff

**Affiliations:** 1 Centre for Internal Medicine, Department of Internal Medicine I, Ulm University Hospital, Albert-Einstein-Allee 23, 89081 Ulm, Germany; 2 Department of Diagnostic and Interventional Radiology, Albert-Einstein-Allee 23, 89081 Ulm, Germany

**Keywords:** Gallbladder polyps, Prevalence, Long-term progression, Ultrasonography

## Abstract

**Background:**

The aim of our study was to investigate the prevalence and natural long-term progression of gallbladder polyps in a random sample of the general population.

**Methods:**

Four hundred and thirteen subjects (190 women, 223 men; aged 29–75 years) were studied first in 2002 and again eleven years later in 2013. All subjects were interviewed using a standardised questionnaire, anthropometric data were recorded, and an abdominal ultrasound scan was carried out.

**Results:**

The prevalence of gallbladder polyps was 6.1% (115/1880) in the 2002 study and 12.1% (50/413) in the 2013 follow-up study. After eleven years, 36 subjects (8.7%, 36/413) had developed new polyps, thirteen subjects (48.1%, 13/27) no longer had gallbladder polyps, and 14 subjects (51.9%, 14/27) still had polyps. The number of polyps had increased in six of these subjects (43%, 6/14), decreased in a further six (43%, 6/14), and remained unchanged in two (14%, 2/14). The mean polyp size was 4.7 mm (± 2.2 mm, range 2–20 mm) in 2002 and 4.0 mm (± 1.9 mm, range 0.5–11 mm) at follow-up. A decrease in polyp size was noted in seven (50%) of the 14 subjects, an increase in size in five subjects (35.7%), and no change in two subjects (14.3%). The shape of the polyps had changed from pedunculated to sessile in two subjects (14.3%, 2/14) and from sessile to pedunculated in one subject (7.1%, 1/14).

**Conclusions:**

In long-term follow-up, the prevalence of gallbladder polyps increased, with new lesions developing in 8.7% of the population. Polyps persisted in 51.9% of the subjects who had them in the original study and disappeared in the other 48.1%.

## Background

Gallbladder polyps are a common incidental finding on abdominal ultrasonography [1, 2]. The growth and management algorithm of small polyps measuring less than 10 mm remained unclear for a long time [1, 3, 4]. In 2017 there were established current guidelines on the treatment and follow-up of gallbladder-polyps [5]. Earlier studies have given very different figures for prevalence, ranging from 0.32 to 26.5% [6, 7]. This discrepancy can be attributed mainly to the differences in study populations and study designs, which greatly restrict the possibilities of comparison. Ultrasound studies on random population samples in Germany have given prevalences of 1.4 and 6.1% [8, 9].

At the present time, only a limited number of studies addressing the long-term progression of gallbladder polyps in non-selected populations are available [2, 3, 7–20]. One follow-up study in a random population sample over an observation period of 30 months showed that 81% of the polyps did not change in size, while 14% had increased and 5% had decreased. At follow-up, gallbladder polyps were no longer demonstrated in 23% of the subjects [8]. After 84 months, 77% of the polyps investigated were the same size, while 8% had become smaller [8].

The aim of our study was to investigate the prevalence and natural long-term progression of gallbladder polyps in a random sample of the general population.

## Methods

**Fig. 1:**
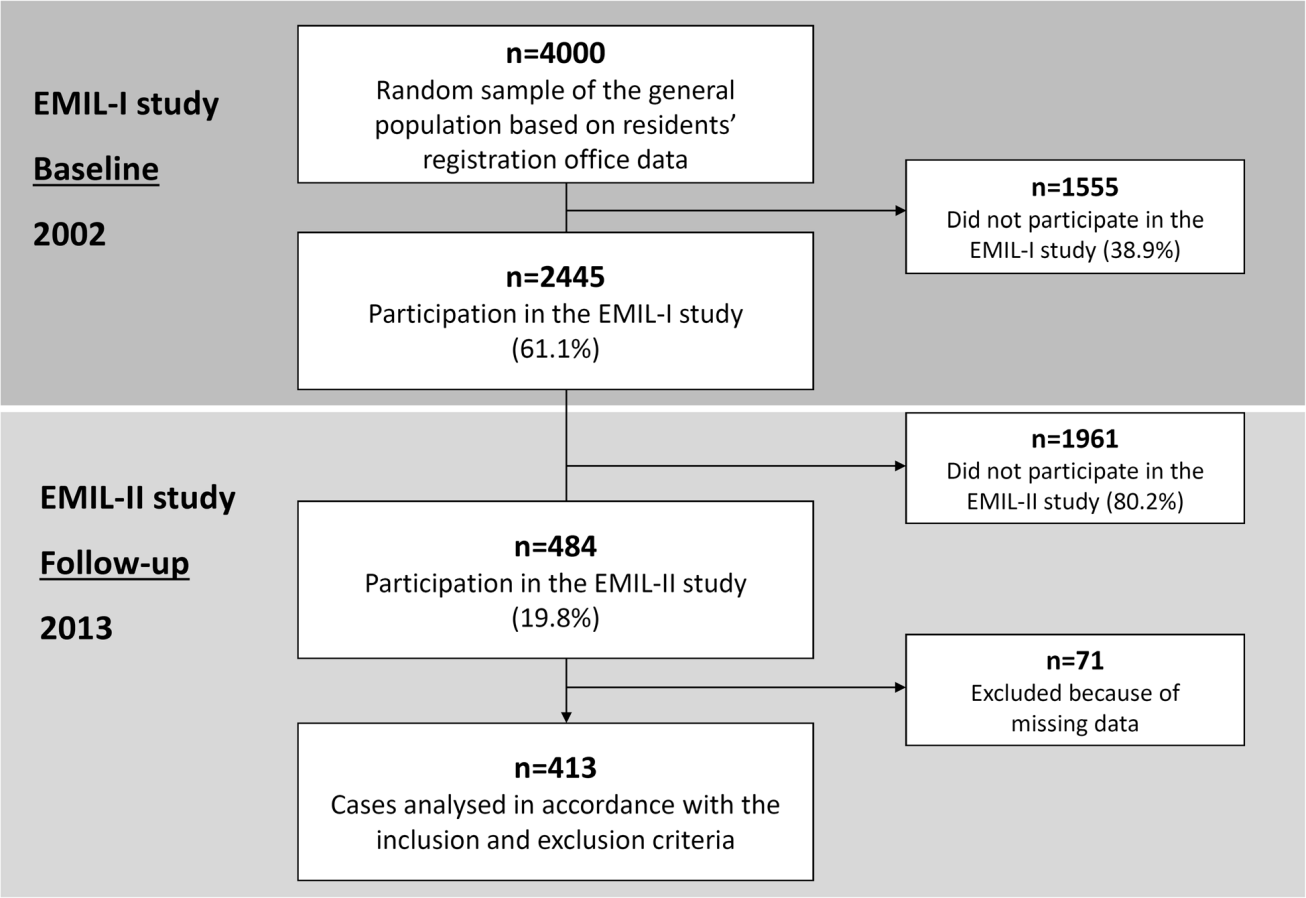
Flow chart showing the inclusion and exclusion of subjects in the gallbladder polyp study from the random sample of the general population (EMIL-I study in 2002 and follow-up EMIL-II study in 2013)

The original *Echinococcus multilocularis* in Leutkirch (EMIL) study in the general population was carried out in 2002 [21]. In that year, 4000 people aged between 10 and 65 years randomly selected from the residents’ registration office in Leutkirch were contacted for the study, of whom 2445 subjects formed the study population. In 2013, following data analysis and matching cases and controls according to gender, age, body mass index (BMI), and the presence or absence of hepatic steatosis, we recruited a subpopulation of 484 out of the original EMIL subjects for the follow-up study. After persons with incomplete datasets had been excluded, we ultimately had an EMIL subpopulation of 413 subjects aged between 29 and 75 years taking part in the follow-up study (Fig. 1). Participation was voluntary. The follow-up study population consisted of 190 (46%) women and 223 (54%) men (Table 1). Using a standardised questionnaire, we asked the subjects about personal details, leisure activities, past medical history, dietary habits, smoking, alcohol consumption and recreational drug use, and family history. Height and weight were measured on the spot. Each subject had an ultrasound scan of the upper abdomen. The gallbladder was measured in three planes, the wall was described as unremarkable or thickened (thickening > 3 mm), and the lumen examined for sludge, stones and polyps. If stones or polyps were detected, we documented the number, site, maximum size in three planes, presence of acoustic shadowing, echogenicity, shape, and contours. Further examinations such as computed tomography scanning or histological analysis have not been performed. It isn’t known whether subjects underwent cholecystectomy in the course.

**Table 1:** Subject characteristics (*n* = 413)

*n* = 413	*n* (%)	Mean ± SD (min-max)
Gender		
Men	223 (54.0%)	
Women	190 (46.0%)	
Age		57.8 ± 11.7 (31.0–74.0)
ns	3 (0.7%)	
18–30-year-olds	0 (0.0%)	
31–40-year-olds	23 (5.6%)	
41–50-year-olds	96 (23.2%)	
51–65-year-olds	167 (40.4%)	
> 65-year-olds	124 (30.0%)	
Body mass index (BMI**)**		29.2 ± 4.3 (20.7–41.1)
Gallbladder polyps		
Total	50/413 (12.1%)	
Men	27/223 (12.1%)	
Women	23/190 (12.1%)	
Polyp size		
Baseline 2002 (EMIL-I)		4.7 ± 2.2 mm (2.0–20.0)
Follow-up 2013 (EMIL-II)		4.0 ± 1.9 mm (0.5–11.0)

The statistical analysis was performed with SAS Version 9.2. Dichotomous variables as well as variables on ordinal and nominal scales were first presented descriptively. After the presentation of sites and extent of distribution, we calculated the age-specific and gender-specific prevalence rates.

## Results

### Prevalence of gallbladder polyps

**Fig. 2:**
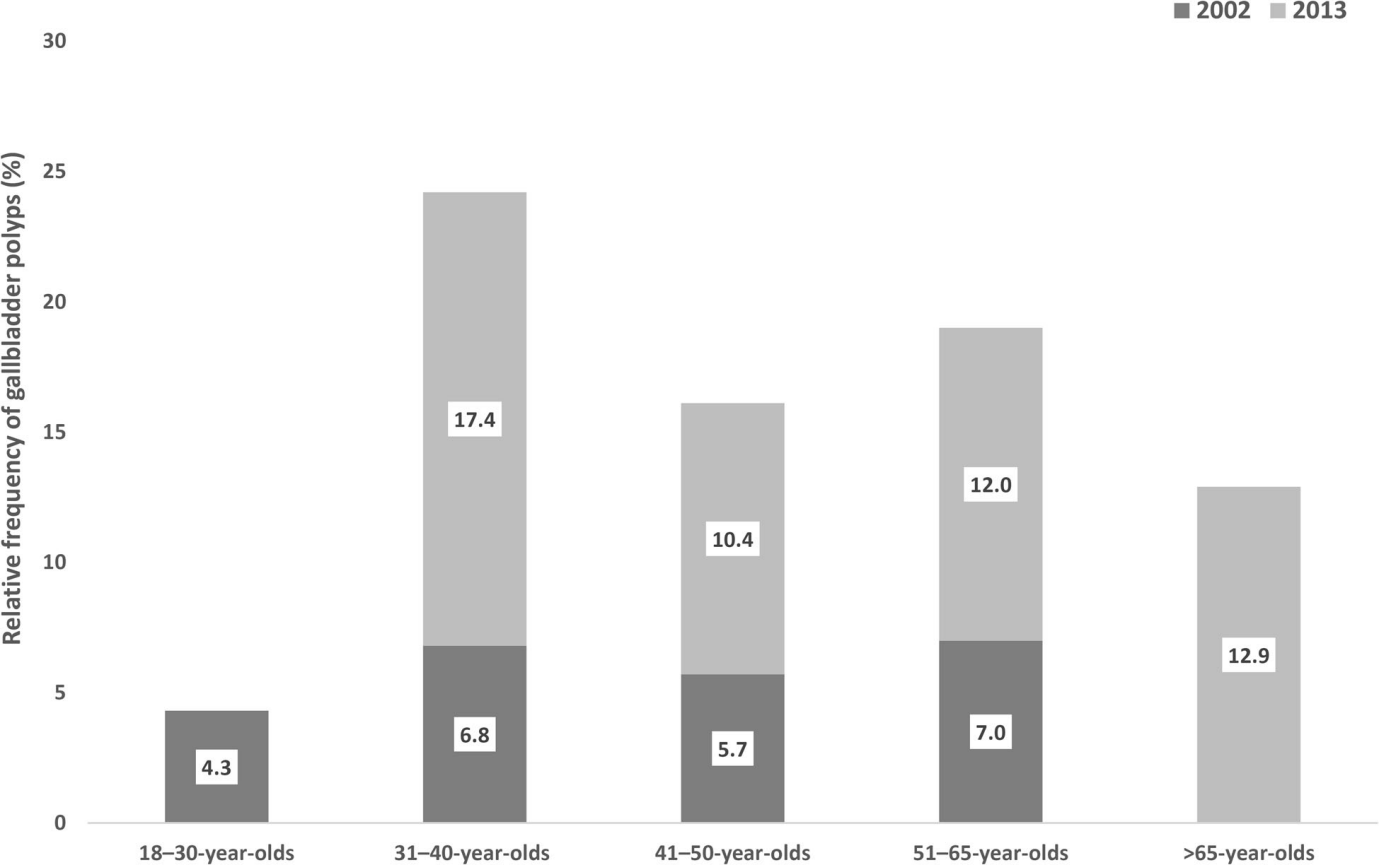
Relative frequency of gallbladder polyps according to age in the original EMIL study in 2002 and the follow-up study in 2013

The prevalence of gallbladder polyps was 6.1% (115/1880 subjects) in the original 2002 study: 6.2% for women and 6.0% for men. There was a trend towards an increasing prevalence of polyps with age, although the rate was already somewhat elevated in the group aged 31–40 (Fig. 2).

In the 2013 follow-up study, the prevalence was 12.1% (50/413 subjects) with women and men being affected in relatively equal proportions (12.1%). The highest prevalence was seen in the 31–40 age group, but there was also an upwards trend with increasing age in the older age groups (Fig. 2). The mean age of the cohort (*n* = 413) was 57.8 ± 11.7 years. The youngest person was 31 years of age and the oldest 74. There were 23 subjects (5.6%) in the 31–40 age group and 124 (30.0%) in the over-65 s. Most of the patients (167; 40.4%) were aged 51–65 years. The mean BMI (body mass index) was 29.2 ± 4.3, ranging from 20.7 to 41.1 (Table 1).

### Progression of gallbladder polyps

In the 2013 follow-up study, we examined 27 subjects who had already had gallbladder polyps in 2002. Fourteen of these subjects (51.9%) still had polyps, while 13 subjects (48.1%) no longer had any evidence of gallbladder polyps on ultrasound scanning. In addition, 36 subjects (8.7%) who did not have polyps in 2002 had developed them by 2013.

The natural progression of gallbladder polyps could be observed in the 14 patients who had polyps in the original study in 2002 and also participated in 2013 (referred to in the following as ‘follow-up subjects’). The number of polyps increased in six subjects (42.9%), decreased in a further six subjects (42.9%), and was unchanged in the remaining two (14.3%). Polyp size decreased in seven subjects (50.0%), increased in five subjects (35.7%), and stayed the same in the other two (14.3%).

### Ultrasound scan characteristics

In general, the majority of polyps were hyperechoic. Over time, the echogenicity had changed from hyperechoic to hypoechoic in one subject. Table 2 shows the observations made in the 14 follow-up subjects with respect to the shape of the polyps. In eight of the 14 subjects (57.1%) the polyps had not changed in shape. The description of the polyps had changed from pedunculated to sessile in two subjects (14.3%) and vice versa from sessile to pedunculated in one subject (7.1%). In two cases, polyps identified in 2013 were described as pedunculated, although it had not been possible to assess them in the original 2002 study. One polyp, reported as pedunculated in the original study could not be assessed in 2013. The gallbladder polyps mostly showed regular contours but had become irregular in two of the subjects in the follow-up group.

**Table 2:** Progression of the gallbladder polyps within the follow-up group (14 patients with gallbladder polyps on examination in 2002 and 2013)

*n* = 14		
Shape in 2002	Shape in 2013	Number (%)
–	unchanged	8 (57.1%)
pedunculated	sessile	2 (14.3%)
sessile	pedunculated	1 (7.1%)
could not be assessed	pedunculated	2 (14.3%)
pedunculated	could not be assessed	1 (7.1%)

## Discussion

In comparison with other population-based studies, the EMIL follow-up study in 2013 showed a higher prevalence (12.1%) of gallbladder polyps. Most of the earlier population-based studies come from Asia [22–27]. Population-based studies from Germany have so far yielded prevalences of 1.4% (incidental findings when ascertaining the prevalence of gallbladder stones) and 6.1% [8, 9]. In Europe, apart from these German studies, only Jørgensen in Denmark has determined population-based prevalences of 4.6 and 4.3% in the up to 60-year-old men and women, respectively, and of 5.9 and 5.8% in 70-year-old men and women [28, 29]. Comparing the population-based studies worldwide, a general increase in the prevalence can be seen over the years from 1990 to date: Jørgensen (1990) 4.6%/4.3%, Segawa (1992) 5.6%, Chen (1997) 6.9%, Okamoto (2002) 8.1%, Kratzer (2010) 6.1%, Park (2013) 8.5% and EMIL (2013) 12.1% [9, 22, 24–29]. In the first instance, this increase may be due to improvements in the ultrasound technology.

Apart from the general population-based studies, there have been many studies on selected populations, for example, during healthcare screening [16, 27, 30–32], oil industry workers in China [33], officers aged between 48 and 56 years in Japan [34], and patient populations [2, 6, 15, 35–37]. While population-based studies have given prevalences in the range of 1.4 to 8.5% – and 12.1% in the EMIL follow-up study – studies on selected subjects have given values between 6.1 and 12.3%, while studies on patient populations have given values of 0.06 to 26.5%. The majority of the values obtained have been below 7% [2, 6–9, 12, 14–16, 22–31, 33–66]. The large differences in the results may be attributed in part to different study designs and methods. Previous prevalence data obtained by ultrasound ranged from 0.32 to 9.96%; data obtained by surgery and pathology were between 0.06 and 21.3% and tended to be somewhat higher than the ultrasound data [2, 6, 8, 9, 12, 15, 16, 22–31, 33–66]. This deviation may demonstrate the fact that not all existing polyps were detected on ultrasound scans in the past, as the technology was not so highly developed. The results of the EMIL follow-up, with 12.1%, are in the same order of magnitude as the surgical/pathological results. For example, Toda et al. found a polyp prevalence of 14.8% and the study by Furukawa et al. gave a corresponding figure of 10.4% [42, 63].

In the EMIL study, we did not find a gender-specific difference in the prevalence of polyps. Csendes and co-workers, as well as Cantürk et al. also found the prevalence to be similar in men and women [3, 35]. In our study, the 31–40 age group showed the highest prevalence at 17.4%. In the original study, too, the group aged 31–40 at that time had a higher prevalence than the following age group. Lin and co-workers reported an increased frequency of gallbladder polyps in 41 to 50-year-olds [31]. Hayashi et al. found the highest prevalence in 40 to 49-year-old men and 50 to 59-year-old women [30]. The presence of gallbladder polyps therefore particularly affects middle-aged people. One explanation for the reduced prevalence of polyps in the older age groups compared with the middle-aged group may be the simultaneous occurrence of gallstones, which possibly leads to the automatic removal of the polyps [32, 35].

In total, 27 subjects with gallbladder polyps from the original study in 2002 were also examined in 2013: polyps were still present in 14 (51.9%) of these subjects but were no longer to be found in 13 (48.1%). Corwin et al. determined a similarly high proportion of polyps that had disappeared (34%) in their follow-up study after 65 months [13]. One German study showed that 22.6% of the polyps had disappeared after 30 months [8], while the follow-up study by Csendes et al. found that 18% of gallbladder polyps could no longer be demonstrated after 48 months [3].

The mean size of the polyps in our 14 follow-up-subjects was slightly larger in 2013 (4.7 ± 1.9 mm) than in 2002 (4.1 ± 1.1 mm) [9]. Collett and Choi et al. also published results showing a maximum growth of about one millimetre [12, 32]. Just as fewer polyps (35.7%) had increased in size and a greater proportion (50%) had reduced in size in the EMIL study, studies carried out in England by Cairns et al. and Corwin et al. showed a similar picture [10, 13]. Most other follow-up-studies have shown that the diameter does not change in the majority of polyps. For example, Park et al. found 75% unchanged, 15% enlarged and 10% decreased in size [16]. Colecchia and co-workers reported polyps that were 91% unchanged, 5.7% enlarged and 3.8% decreased in size [11]. In contrast, Wolpers published findings showing that in subjects with multiple polyps only 11% remained unchanged after a period of about seven years: 28% had grown and 13% had shrunk [7]. The EMIL follow-up study in 2013 also showed a higher proportion of multiple gallbladder polyps. These correlations allow us to conclude that the small number of subjects with unchanged gallbladder polyps could simply be due to the larger number of polyps per subject.

At the end of the follow-up period in the EMIL study, the number of gallbladder polyps was the same in only two subjects (14.3%). Half of the remaining follow-up subjects had more (42.9%) gallbladder polyps in 2013 while the other half (42.9%) had fewer polyps. Other follow-up-studies – provided that data on the number of polyps over time are available – have shown an increase rather than a decrease in the number of polyps [3, 7, 10]. Wolpers, for example, reported an increase in number in 21% of subjects with multiple polyps compared with a reduction in number in 14% [7]. After 48 and 96 months, Csendes et al. found an increase in 20 and 24% of subjects, respectively, while the corresponding figures for a reduction in the number of polyps were just 6 and 3% [3].

Observation of the gallbladder polyps with respect to echogenicity, shape, and contours showed very little change with time. Echogenicity changed from hyperechoic to hypoechoic in only one subject, and the previously regular contours became irregular in two subjects.

The polyps changed from pedunculated to sessile in two of the 14 subjects and, vice versa in three cases, with polyps becoming pedunculated which had previously been sessile or impossible to assess.

These changes may be attributed to improvements in ultrasound technology with greater resolution. Our follow-up population is too small for us to make more precise statements on the relevance of these changes. Previous follow-up studies on gallbladder polyps have not recorded any data on changes in echogenicity, shape or contours with time, so that we are unable to say more at the present time [2, 3, 7–20].

## Conclusions

In our 2013 follow-up study, we found the prevalence of gallbladder polyps to be considerably higher than in the original 2002 study. Looking at other studies as well, there has been a progressive increase in the prevalence over the years from 1990 to the present. One possible reason for the increase may be improvements in ultrasound technology. In the long term, new polyps developed in 8.7% of our study population, existing polyps persisted in 51.9% of subjects and disappeared in 48.1%. The improved ultrasound technology seems to be the reason for the higher prevalence. We have confirmed a higher prevalence in middle-aged subjects.

## Abbreviations

%: percent
BMI: Body mass index
EMIL: Echinococcus multilocularis in Leutkirch
mm: millimetre
SAS: Statistical analysis system
